# The m6A methyltransferase METTL5 promotes neutrophil extracellular trap network release to regulate hepatocellular carcinoma progression

**DOI:** 10.1002/cam4.7165

**Published:** 2024-04-12

**Authors:** Qi Wang, Yuxi Huang, Yu Zhu, Wenlong Zhang, Binfeng Wang, Xuefeng Du, Qiqiang Dai, Fabiao Zhang, Zheping Fang

**Affiliations:** ^1^ Department of Hepatobiliary Surgery, Taizhou Hospital of Zhejiang Province Wenzhou Medical University Linhai Zhejiang China; ^2^ Department of Hepatobiliary Surgery, Taizhou Hospital of Zhejiang Province Linhai Zhejiang China

**Keywords:** hepatocellular carcinoma, immune infiltration, m6A, METTL5, prognostic

## Abstract

**Background:**

Hepatocellular carcinoma (HCC) is one of the most common malignant tumors worldwide, it has a poor prognosis due to its highly invasive and metastatic nature. Consequently, identifying effective prognostic markers and potential therapeutic targets has been extensively investigated. METTL5, an 18S rRNA methyltransferase, is abnormally high in HCC. But its biological function and prognostic significance in HCC remain largely unelucidated. This study aimed to investigate the role of METTL5 in HCC progression, and elucidate its possible molecular mechanisms in HCC via transcriptome sequencing, providing new insights for identifying new HCC prognostic markers and therapeutic targets.

**Methods:**

The METTL5 expression in HCC and paracancerous tissues was analyzed using HCC immunohistochemical microarrays and bioinformatic retrieval methods to correlate METTL5 with clinicopathological features and survival prognosis. We constructed a METTL5 knockdown hepatocellular carcinoma cell line model and an animal model to determine the effect of METTL5 on hepatocellular carcinoma progression. Subsequently, RNA sequencing was performed to analyze the molecular mechanism of METTL5 in HCC based on the sequencing results, and relevant experiments were performed to verify it.

**Results:**

We found that METTL5 expression was elevated in hepatocellular carcinoma tissues and correlated with poor patient prognosis, and in the analysis of clinicopathological features showed a correlation with TNM staging. In hepatocellular carcinoma cell lines with knockdown of METTL5, the malignant biological behavior was significantly reduced both in vitro and in vivo. Based on the sequencing results as well as the results of GO functional enrichment analysis and KEGG pathway enrichment analysis, we found that METTL5 could promote the generation and release of neutrophil extracellular capture network (NETs) and might further accelerate the progression of HCC.

**Conclusion:**

The m6A methyltransferase METTL5 is overexpressed in hepatocellular carcinoma (HCC) and correlates with poor prognosis. METTL5 accelerates malignant progression of HCC by promoting generation and release of the neutrophil extracellular traps (NETs) network, providing new insights for clinical biomarkers and immunotherapeutic targets in HCC prognosis.

## Graphical abstract

Hepatocellular carcinoma (HCC) overexpresses the N6‐methyltransferase METTL5 which correlates with malignant cell behavior and poor prognosis. Furthermore, METTL5 overexpression may induce NET release, influencing the tumor immune microenvironment and promoting distant cancer cell metastasis. Accordingly, we suggest that METTL5 as an immunotherapeutic and prognostic marker is promising for HCC.

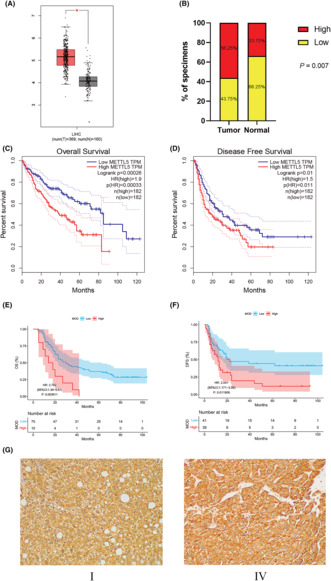

## 1 INTRODUCTION

Liver cancer has emerged as a major health challenge globally.^1^ By 2025, it is projected to affect over one million people annually.^2^ It is thought that most liver cancers are caused by hepatocellular carcinomas (HCC).^3^ HCC pathogenesis is considered a complex, multistep, multifactorial process influenced by environmental and dietary factors.^3^ Despite recent advances in HCC treatment, patient prognosis remains unsatisfactory due to HCC's highly metastatic and invasive nature.^4^ Thus, identifying effective early tumor markers and therapeutic targets are urgent tasks for HCC patients.

N6‐methyladenosine (m6A) modification can regulate multiple RNA‐related processes, including RNA stability,^5^ translation,^6^ and alternative splicing.^7^, ^8^ The modification of m6A can regulate reversible dynamic epigenetics of many RNA biological processes: “writers”, “erasers,” and “readers” are epigenetic modulator enzymes that control their activity.^9^ Several m6A‐modified genes have been reported to be involved in tumourigenesis, infiltration, and metastasis. For instance, YTHDF2, METTL3, and FTO have been shown to be involved in tumourigenesis and development of tumors such as lung, colorectal, breast and gastric cancers, and to affect patient prognosis.^10^, ^11^, ^12^, ^13^

It has only been recently that METTL5 has been extensively studied in the METTL family. An m6A‐catalyzed NPPF motif is conserved in METTL5, along with typical GxGxG motifs. There have been shown to be a multispecies 18S rRNA m6A‐modified methyltransferase^14^, ^15^, ^16^ and regulate ribosome function and development.^17^, ^18^ A previous study demonstrated that METTL5 loss reduces the translation rate of mouse embryonic stem cells (MESCs), impairs spontaneous pluripotency, and impairs differentiation ability.^19^, ^20^ In addition, in breast cancer, METTL5 can promote the initiation of RNA translation and cell growth. In lung adenocarcinoma, METTL5 expression is elevated and associated with poor prognosis.^15^ As of yet, a clear understanding of METTL5's role in HCC is still elusive.

### 1.1 Data and software

The Cancer Genetic Atlas (TCGA) of HCC patients (https://www.cancer.gov/ccg/research/genome‐sequencing/tcga), LIHC data from the University of California, Santa Cruz Xena (https://xenabrowser.net/datapages/) were obtained.

HCC METTL5 expression analysis: Based on immunohistomicroarray analysis of METTL5 expression in 80 patients with HCC, different clinical stages (TNM staging, grading, and staging) were analyzed using the Kruskal–Wallis test.

### 1.2 Survival analysis of METTL5 in HCC

The 80 patients in the immunohisto‐microarray were divided into high‐ and low‐expression groups. In survival analysis, the “survminer” software package was used to analyze the difference in survival between high‐ and low‐expression groups based on METTL5 expression value, survival time, and survival status, then analyzed the difference in survival between groups using the Kaplan–Meier test.

### 1.3 Cell culture

HCC cells HepG2, SMMC‐7721, and MHCC‐97H, the normal hepatocyte cell line LO2, and HL‐60, these were purchased from the Chinese Academy of Sciences Stem Cell Bank (Shanghai). FBS‐supplemented RPMI‐1640 medium was used to culture SMMC‐7721 cells. In addition, 10% FBS was added to DMEM medium to culture the remaining cells. Incubation with 5% CO_2_ at 37°C resulted in the absence of mycoplasma in all cells, and the cells were identified using STR analysis.

### 1.4 qRT‐PCR

Using Trizol (Ambion, Invitrogen, USA) RNA extraction kit, we extracted RNA from cells. The cDNA was synthesized using the PrimeScript RT Reagent Kit (Takara, Cat: RR037A, Dalian, China), followed by detection using TB Green Premix Ex Taq (Takara, Cat: RR820A, Dalian, China) on the ABI PRISM 7500 Sequence Detection System (Applied Biosystems, Inc.). The obtained results were calculated using the 2^−ΔΔct^ method with GAPDH as the internal control. The qRT‐PCR primer sequences used were synthesized by RIBOBIO (Guangzhou, China) and are shown below.
METTL5: Forward: 5'-AAGGAACTAGAGAGAGTCGCC  TG-3'.
 Reverse: 5'-GCGGCCTGGGTAGGATACTG-3'.
GAPDH: Forward: 5'-ATAGCACAGCCTGGGATAGC  AACGTAC-3'.
 Reverse: 5'-CACCTTCTACAATGAGCT  GCGTGTG-3'.



### 1.5 Western blot

Use RIPA lysis buffer to extract cell proteins, add the proteins onto a 10% SDS‐PAGE gel for electrophoretic separation, then transfer to a PVDF membrane. Block the membrane with 5% skimmed milk for 2 h, wash with TBST solution, and incubate with primary antibodies (GAPDH (Proteintech, 1E6D9, 36KDa), METTL5 (Proteintech, 16791‐1‐AP, 27KDa), and PADI4 (Proteintech, 17373‐1‐AP, 65KDa)) 4°C for 12–14 h. Then, incubate with secondary antibodies, and detect protein bands using enhanced chemiluminescence reagent. The results were calculated using Image J software (USA, version 1.6.0). GAPDH was used for normalization.

### 1.6 Lentivirus construction and cell transfection

The lentiviral vector used in the METTL5 gene knockdown group and blank control group was purchased from GENE (Shanghai, China). Appropriate amounts of viral solution were added to HepG2 and SMMC‐7721 cell lines according to different viral titers. We added puromycin at a concentration of 2 μg/mL to the cell lines after they expressed GFP fluorescence and then screened them for 48–72 h. Stable METTL5 knockdown and blank control cell lines were obtained for subsequent experiments, which were subsequently verified by western blot and qRT‐PCR.

### 1.7 RNA sequencing detection and analysis

Lentiviral stable‐transfected strains with high METTL5 knockdown efficiency and negative control cells were selected, and total RNA was extracted after amplification. Concentration and purity were assessed by spectrophotometry, and transcriptome sequencing was performed by RIBOBIO (Guangzhou, China) for three samples each from the METTL5‐KO and METTL5‐NC groups.

### 1.8 CCK‐8 assay and plate cloning assay

In 96‐well plates, 1000 cells of HepG2 and SMMC‐7721 were seeded and cultured for 24, 48, 72, and 96 h in an incubator. The CCK‐8 assay reagent (Beyotime, Cat. No. C1052) was used to determine cell viability after 24, 48, 72, and 96 h, and the absorbance value at 450 nm was measured using an enzyme‐linked immunosorbent assay (ELISA) reader (Thermo). To determine the colony‐forming ability of the cells, 1000 cells per petri dish were counted and cultured in an incubator for 14 days. To determine statistical significance, the colonies were photographed and counted after being fixed and stained with paraformaldehyde for 20 min.

### 1.9 Scratch healing experiment

HepG2 and SMMC‐7721 cells from control and experimental groups were inoculated into 6‐well plates. We made artificial wounds using a 200 μL pipette tip after even spreading. For 24 h, the cells were incubated in medium with 1% FBS after being washed with PBS. Using a microscope camera, 0‐and 24‐h photographs were taken at 100× magnification (Thermo) to evaluate scratch healing.

### 1.10 Transwell migration and invasion assays

A 24‐well plate was used for experiments with and without matrix gel (BD Biosciences, USA) in small chambers (8.0 um pore size, 6.5 mm diameter). To the upper chamber, 100 μL of each cell type was added in serum‐free medium at a concentration ranging from 2 to 3.5 × 10^5^/mL. The lower chamber was filled with medium containing 10% FBS. We incubated the cells for 24 h then removed from chambers, fixed with 4% paraformaldehyde, the upper chamber was stained with crystal violet 0.1% and gently wiped with cotton swabs. To determine the cell number, different fields of view were used to photograph cells randomly.

### 1.11 Apoptosis

When cells of the experimental and control groups reached 70%–80% confluence, they were trypsinized and collected, then stained with Annexin V‐APC/7‐AAD (Elabscience) Apoptosis Detection Kit. Apoptosis was detected in samples by flow cytometry (BD, FACSMelody, USA).

### 1.12 Subcutaneous tumor formation experiments in nude mice

Mice studies were conducted according to the guidelines of Taizhou Hospital's Animal Experimentation Ethics Committee. BALB/C‐nu mice (6 weeks old, Hangzhou Paisio Biotechnology Co., Ltd.) were reared under specific pathogen‐free (SPF) conditions, and resuspended cells of different subgroups (1 × 10^6^ cells/cell) were inoculated into the right subcutaneous side of each nude mouse. The right side of the nude mice was observed every 3 days and the tumor diameter was measured. There were mice euthanizsed with CO_2_ when they showed severe discomfort or after 3 weeks of tumor formation, and the tumors were isolated for subsequent experiments, including immunohistochemistry and immunofluorescence.

### 1.13 Immunohistochemical Staining and Immunohistofluorescence

IHC (Immunohistochemical Staining Kit, Cell Biological, China) and H&E (H&E Staining Kit, Solarbio, China) were performed on paraffin‐embedded tumor tissues.

Immunofluorescence: Dewaxed, antigen‐retrieved, and sealed paraffin‐embedded mouse HCC tissue sections, a primary antibody Cit H3 (1:100) (HUABIO, ET1701‐64) and a secondary antibody MPO (1:100) (Abcam, Lot: 1000224‐2) were incubated overnight with cells, following three washes with PBS, the secondary antibody was incubated three times. After incubation for 1 h, washed again with PBS, then stained with DAPI, and finally blocked with anti‐fluorescence quencher. The tissue sections were observed under a laser confocal microscope.

### 1.14 Cell coculture and cell immunofluorescence

HepG2 and SMMC‐7721 cells from METTL5‐NC and METTL5‐sh groups were digested with trypsin and suspended at 1 × 10^5^ cells/mL. The cells were inoculated into a 24‐well plate. After cell adhesion, the cells were transferred to transwell chambers. The HL‐60 cells were inoculated into the chambers and a coculture system was established. The upper layer of the cells were collected from the chambers after 3 days of cultivation for subsequent experiments.

The HL‐60 cells were collected, evenly coated onto microscope slides, and fixed for 30 min with 4% paraformaldehyde. After washing with PBS after permeabilization with 0.5% Triton X‐100 for 10 min, and incubation with phosphate buffer containing 10% FBS for 30 min, the cells were incubated 12–14 h with MPO (1:100) and Cit H3 (1:100). The second antibiotic resistance was incubated for 1 h and DAPI staining dye was applied for 10–20 min.

### 1.15 Statistical analysis of data

Our statistical analyses were conducted using GraphPad Prism (version 9.0), R (version 4.03), and Rstudio (version 1.2.5042). As stated, each experiment was repeated three times, and statistical significance was determined at *p* ＜ 0.05. *P ＜ 0.05, **P ＜ 0.01, ***P ＜ 0.001.

## 2 RESULTS

### 2.1 Expression of METTL5 in HCC and liver tissue

**FIGURE 1:**
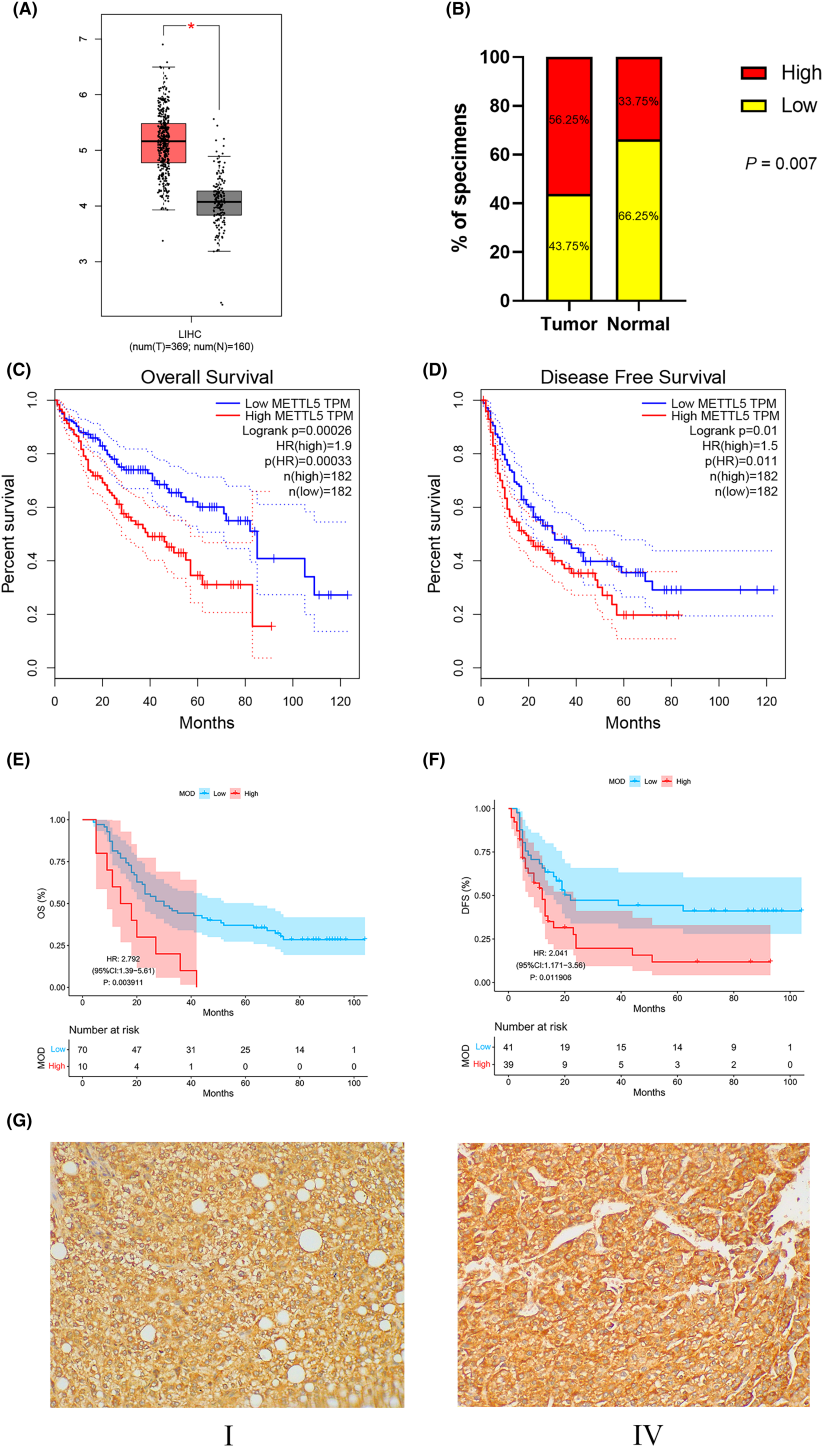
Analysis of METTL5 expression in HCC using immunohistochemistry and TCGA‐LIHC data. HCC and normal liver tissue METTL5 expression based on LIHC (unpaired samples in A), and expression in hepatocellular carcinoma and paracarcinoma tissues based on immunohistochemistry (paired samples in B); (C, D) From the TCGA database, Kaplan–Meier curves of METTL5 expression in HCC are shown. (E, F) METTL5 expression levels in HCC based on immunohistochemistry Kaplan–Meier curves. (G) METTL5 expression levels in hepatocellular carcinoma and paracarcinoma tissues at Stages I and IV.

The results of METTL5 expression in cancer and paracancerous tissues based on immunohistomicroarrays of 80 cases showed that the same expression of METTL5 was upregulated in HCC (*p* < 0.01) (Figure 1B), and to assess the effect of METTL5 on survival prognosis, METTL5 expression levels were compared between patients in high‐ and low‐expression groups, results showed that high‐expression patients had a shorter OS and DFS than low‐expression patients (Figure 1E,F).

We analyzed 529 samples based on TCGA‐LIHC to determine METTL5's role in HCC. METTL5 was found to be overexpressed in HCC compared with normal tissue (Figure 1A). A high METTL5 expression was associated with a shorter overall survival (OS) and disease‐free survival (DFS) in HCC patients compared to patients with a low expression (Figure 1C,D).

According to immunohistochemical array results, paracancerous tissues expressed more METTL5 than cancerous ones, and higher in patients with TNM stage IV versus Stage I (Figure 1G). These results suggest METTL5 significantly affects HCC patient prognosis and may have higher validity for predicting HCC prognosis.

As shown in Table 1, multivariate regression analysis of 80 HCC patients by age, sex, AFP, and TNM stage showed METTL5 expression associated with TNM stage (*p* < 0.05): higher stage correlated with higher METTL5 expression (Table 1).

**TABLE 1:** Correlation analysis between METTL5 and clinicopathological factors.

Parameters	Cases (*n* = 80)	METTL5 expression (*n* = 80)		*p*
		Low (*n* = 40)	High (*n* = 40)	
Age (year)				0.749
<57	59 (73.8%)	30 (37.5%)	29 (36.3%)	
≥57	21 (26.2%)	10 (12.5%)	11 (13.8%)	
Gender				0.155
Male	43 (53.8%)	23 (28.8%)	20 (25.0%)	
Female	37 (46.2%)	19 (23.8%)	18 (22.5%)	
AFP				0.540
<7 μg/L	28 (35.0%)	15 (18.8%)	13 (16.3%)	
≥7 μg/L	52 (65.0%)	25 (31.3%)	27 (33.8%)	
TNM stage				**0.041** *
I	26 (32.5%)	11 (13.8%)	5 (6.3%)	
II	18 (22.5%)	13 (16.3%)	6 (7.5%)	
III	19 (23.7%)	10 (12.5%)	11 (13.8%)	
IV	17 (21.3%)	7 (8.8%)	17 (21.3%)	
Metastasis	14 (17.5%)	7 (8.8%)	7 (8.8%)	0.220
Hepatitis B	66 (82.5%)	34 (42.5%)	32 (40.0%)	0.735

* *p* < 0.05.

### 2.2 Regulation of METTL5 expression levels in SMMC‐7721 and HepG2 cells

**FIGURE 2:**
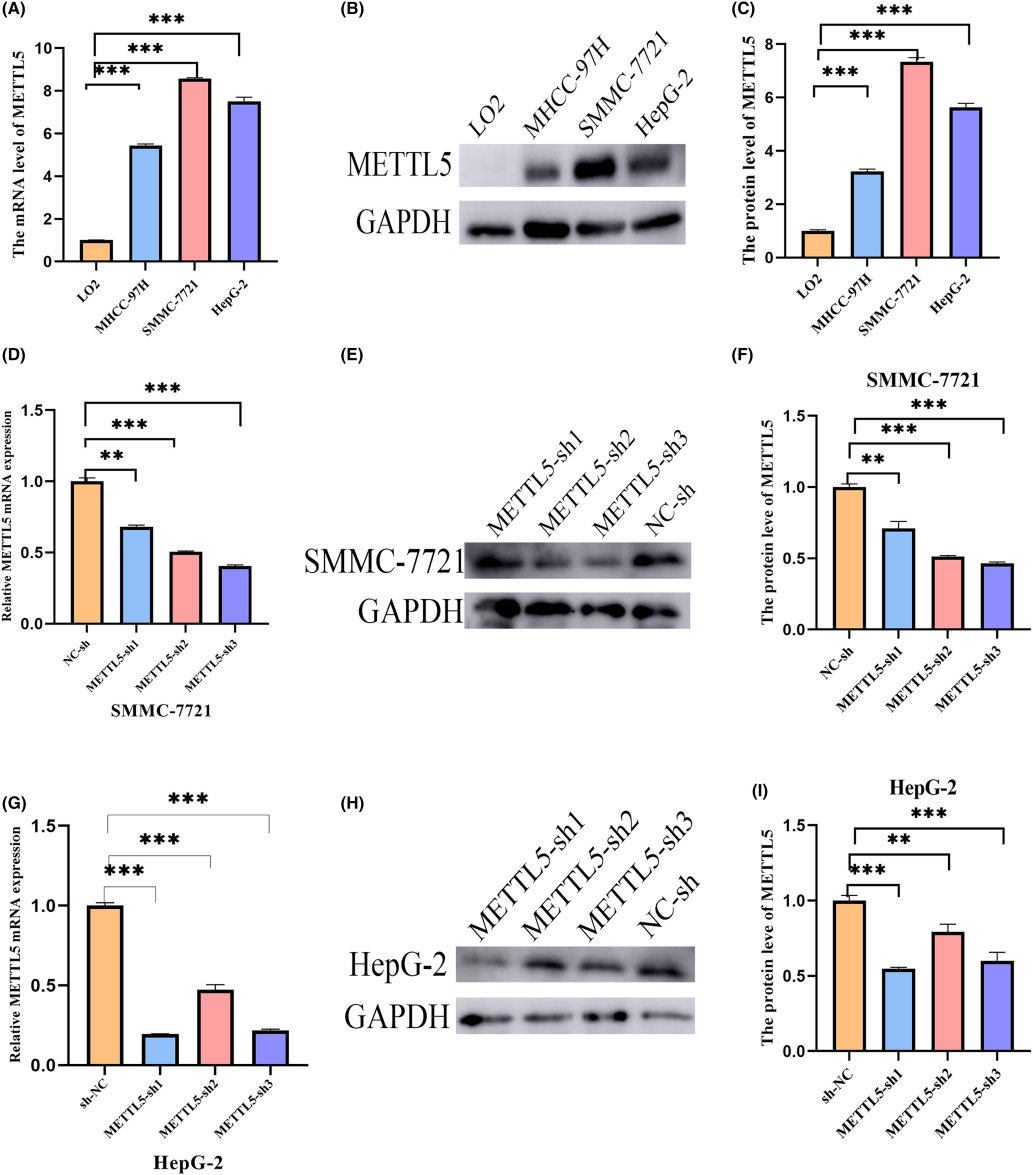
SMMC‐7721 and HepG2 cells express METTL5 in HCC cells and how this affects their biological functions. (A) mRNA expression of METTL5 in LO2 MHCC‐97H, SMMC‐7721, and HepG2; (B, C) Protein expression of METTL5 in LO2 MHCC‐97H, SMMC‐7721, and HepG2; (D–I) Detection of METTL5 mRNA and protein expression in SMMC‐7721/HepG2 cells by qRT‐PCR and western blot. **p < 0.01. ***p < 0.001.

The first step was to validate the expression level of METTL5 in LO2, MHCC‐97H, SMMC‐7721, and HepG2. As compared to human normal liver cells, liver cancer cell lines expressed significantly higher levels of METTL5 (Figure 2A–C). The results indicated that SMMC‐7721 and HepG2 expressed more METTL5; we chose these two cell lines for the upcoming experiments. Lentiviral transfection was used to downregulate the expression of METTL5 in SMMC‐7721 and HepG2. The knockdown efficiency was verified after transfection, and the results showed that the knockdown efficiency of METTL5‐sh3 was best in SMMC‐7721 (Figure 2D–F), and METTL5‐sh1 was best in HepG2 (Figure 2G–I). Cell lines with liver cancer expressed less METTL5 protein after transfection. For the subsequent experiments, stable lentiviral transfections with the highest knockdown efficiency were chosen.

### 2.3 METTL5 affects the biological function of HCC cell lines in the following ways

**FIGURE 3:**
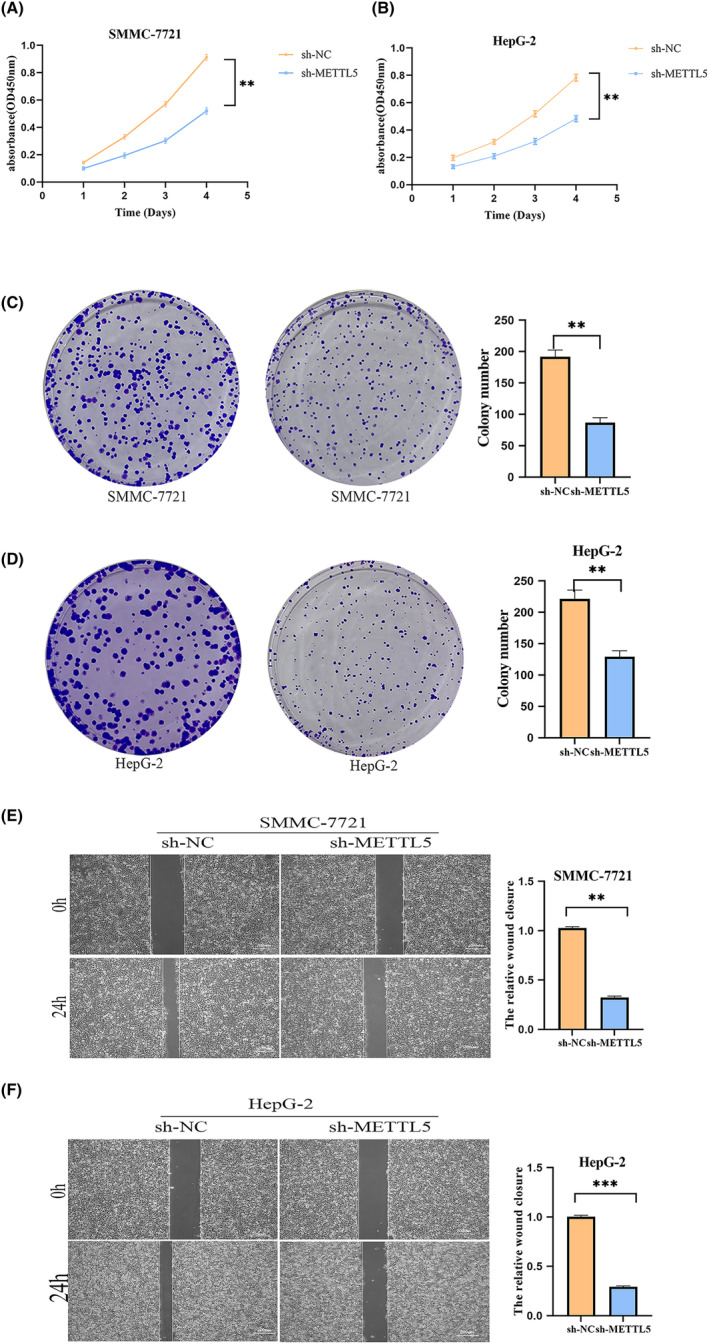
Effect of METTL5 on proliferation and scratching of HCC cells. (A, B) CCK‐8 cell proliferation assay; (C, D) plate cloning assay; (E, F) scratch assay. **p < 0.01. ***p < 0.001.

**FIGURE 4:**
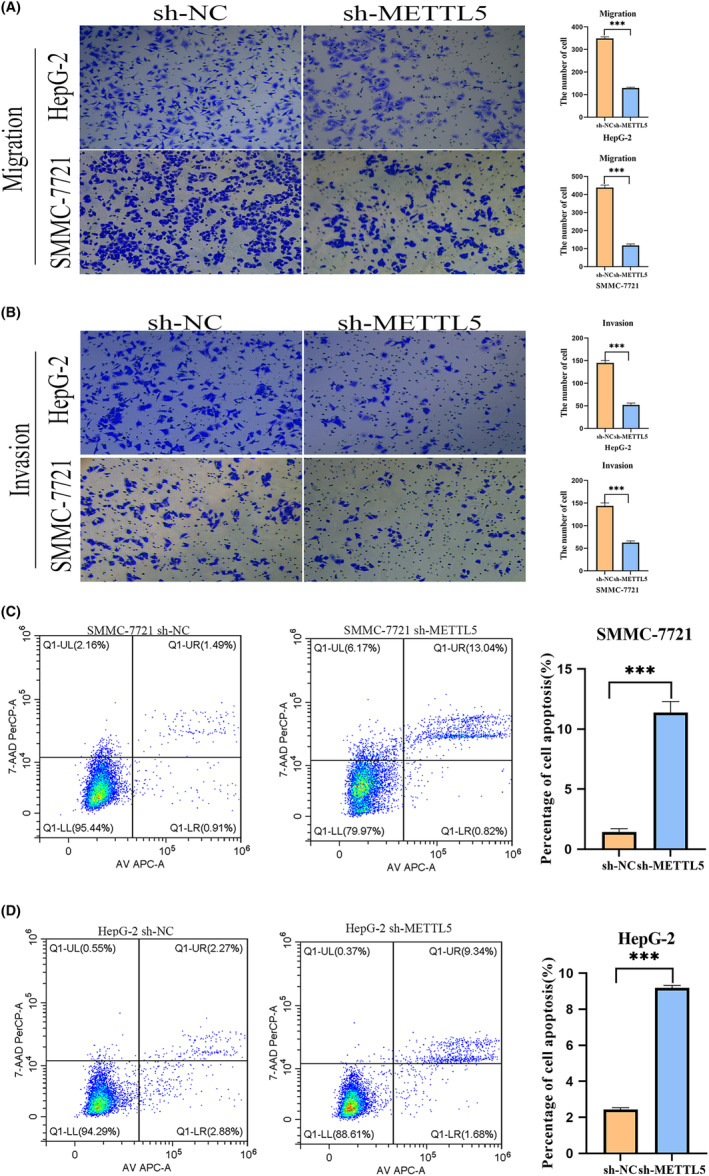
Effect of METTL5 on the biological function of HCC cells. (A, B) Cell migration and invasion assay; (C, D) Flow cytometry for cell death assay. ***p < 0.001.

**FIGURE 5:**
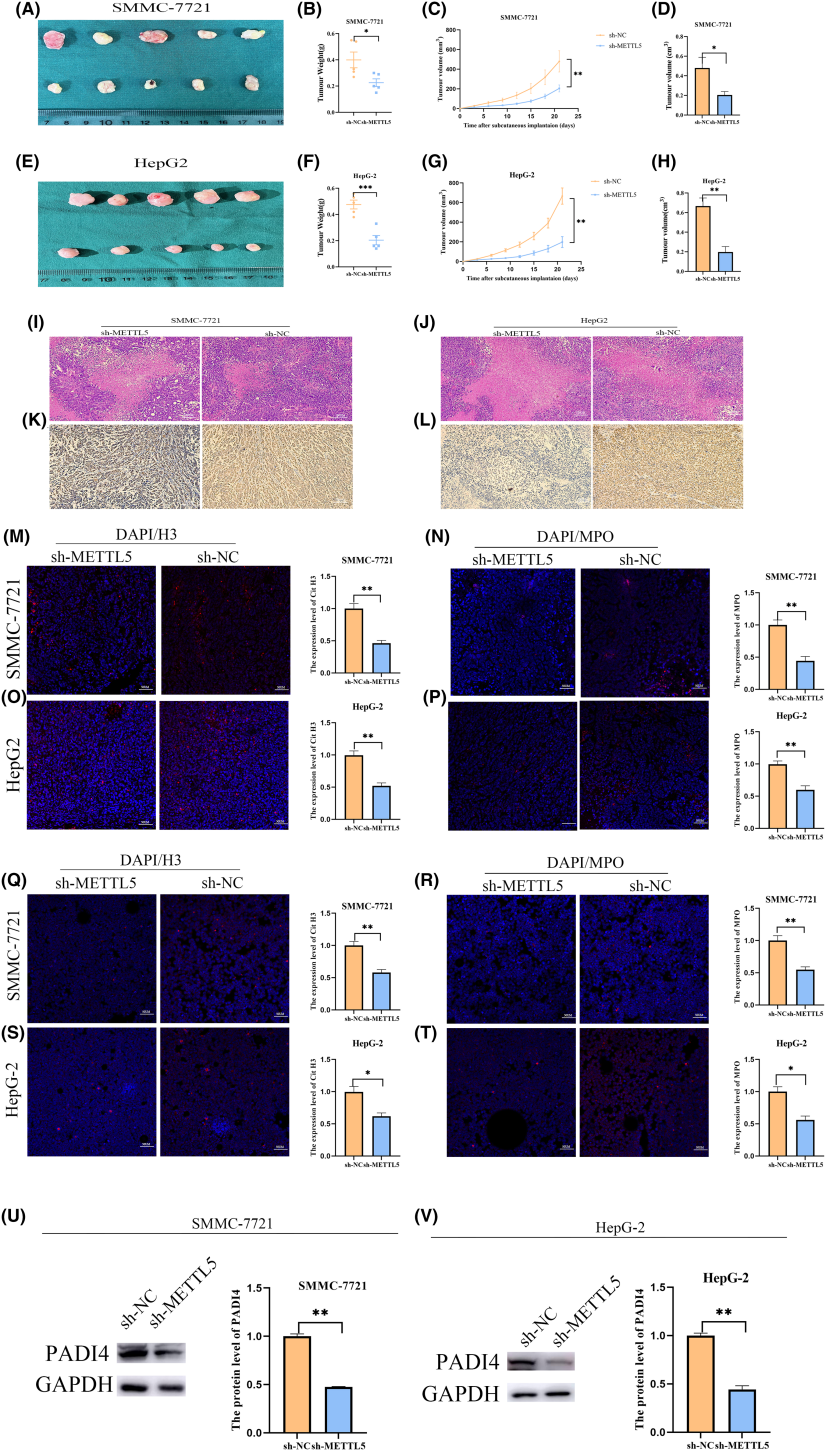
METTL5 affects tumor size, proliferation, necrosis, and NETs in subcutaneous tumorigenesis experiments in nude mice. Subcutaneous tumorigenesis model using SMMC‐7721 cell line: (A, D) tumor volume, (B) tumor weight, and (C) tumor growth curve. Subcutaneous tumorigenesis model using HepG2 cell line: (E, H) tumor volume, (F) tumor weight, and (G) tumor growth curve. (I, J) HE staining of tissue from tumor sections of SMMC‐7721 and HepG2 cell line showing tumor necrosis area. (K, L) SMMC‐7721 and HepG2 cell line‐derived tumor section stained with Ki‐67. (M, O) Tumor section tissue derived from SMMC‐7721 and HepG2 cell lines stained for DAPI/H3 immunofluorescence expression level. (N, P) DAPI/MPO immunofluorescence expression level of tumor section tissues stained with DAPI/MPO derived from SMMC‐7721 and HepG2 cell lines. (Q, S) DAPI/H3 immunofluorescence expression level of HL‐60 cells stained with DAPI/H3 derived from SMMC‐7721 and HepG2 cell lines after coculturing with HL‐60 cell line. (R, T) DAPI/MPO immunofluorescence expression level of HL‐60 cells stained with DAPI/MPO derived from SMMC‐7721 and HepG2 cell lines after coculturing with HL‐60 cell line. (U, V) SMMC‐7721 and HepG2 cell line and HL‐60 cell line cocultured with HL‐60 cell line collected after HL‐60 cells stained with PADI4 protein expression level. *p < 0.05, **p < 0.01. ***p < 0.001.

The cell viability of SMMC‐7721 and HepG2 cells transfected with METTL5‐sh was reduced as detected by CCK‐8 (Figure 3A,B). Plate cloning assay showed that METTL5 knockdown inhibited cloning ability and proliferation of SMMC‐7721 and HepG2 cells (Figure 3C,D). SMMC‐7721 and HepG2 cells were significantly inhibited by METTL5 knockdown in scratch assays (Figure 3E,F). Transwell cell migration and invasion assays result showed that downregulation of METTL5 significantly reduced migration and invasion ability (Figure 4A,B). Finally, flow cytometry detection of cell apoptosis in SMMC‐7721 and HepG2 cells transfected with METTL5‐sh showed increased apoptosis after METTL5 downregulation (Figure 4C,D). Our cellular experiments demonstrated that high METTL5 expression in HCC led to malignant tumor cell behavior and poor patient prognosis. To further validate METTL5's role in HCC, an in vivo subcutaneous tumorigenesis model was established using HepG2 cells grown in SMMC‐7721 and METTL5‐sh cells in nude mice. Tumor growth rate and weight significantly decreased upon METTL5 downregulation versus control (Figure 5A–H). Immunohistochemistry of paraffin‐embedded subcutaneous tumor tissues from nude mice showed larger necrotic areas in tumor sections from both SMMC‐7721 and HepG2 cell lines with METTL5‐sh versus negative control by HE staining (Figure 5I,J). KI‐67 is a proliferation marker and was lowly expressed in METTL5‐sh tumor tissue sections (Figure 5K,L). These results support METTL5's role in HCC tumorigenesis.

### 2.4 To analyze possible mechanisms by which METTL5 affects HCC progression and prognosis

**FIGURE 6:**
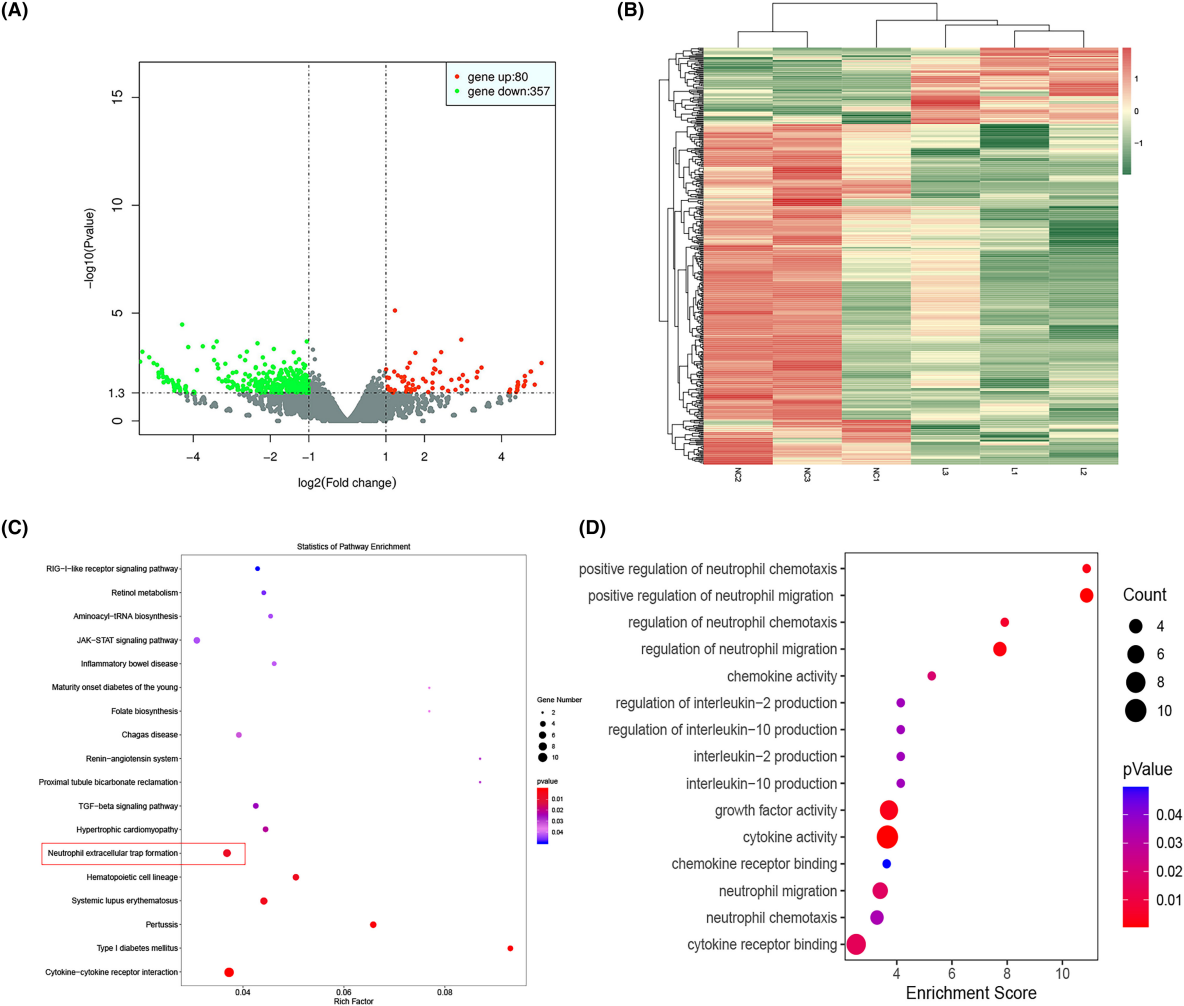
RNA sequencing results. (A) volcano map; (B) heat map; (C) KEEG pathway enrichment analysis; (D) GO function enrichment analysis.

By analyzing RNA sequencing data of METTL5‐sh group and METTL5‐NC, differentially expressed genes were identified in HCC cells compared to the METTL5 knockdown group (Figure 6A,B). Screening of differentially expressed genes in HCC cell lines after METTL5 knockdown by RNA‐Seq and GO functional enrichment analysis of differentially expressed mRNAs also showed that METTL5 significantly increased the generation and release of the extracellular capture network in centromeres (Figure 6D). And subsequent KEGG pathway enrichment analysis showed a significant increase in centriolar extracellular capture network release (Figure 6C). The above results indicate that METTL5 expression is upregulated in HCC and suggest that this poor prognosis may be related to increased NETs release.

### 2.5 Effect of METTL5 on the release of the neutrophil external capture network

In the METTL5‐sh group, MPO and H3 expression were lower than negative control group in subcutaneous tumor tissue sections of nude mice derived from either SMMC‐7721 or HepG2 cell lines (Figure 5M–P). Immunofluorescence results of HL‐60 cells cocultured with HL‐60 cells from METTL5‐sh and METTL5‐NC groups showed MPO/H3 expression in METTL5‐NC group was higher than METTL5‐sh group, consistent with tumor tissue results (Figure 5Q–T). PADI4 expression level in METTL5‐NC coculture group with HL‐60 cells was significantly higher than METTL5‐sh (Figure 5U,V).

## 3 DISCUSSION

m6A is a common modification of mRNA in eukaryotes,^21^ it has been shown that cancer progression is influenced by methylation of m6A,^22^, ^23^ and changes in RNA methylation are abnormal in a variety of malignant tumors, playing the key role in migration, invasion,regulating tumor immunosuppression, and chemotherapy resistance.^24^, ^25^ It has been demonstrated that abnormal modifications to m6A are associated with HCC progression. For example, in HCC, as METTL3 expression increases, SOCS2 undergoes increased m6A modifications to promote tumor development.^26^ METTL14 can inhibit liver cancer metastasis in an m6A‐dependent manner by increasing miR126 levels.^27^ This is consistent with our research findings, indicating that METTL5 also promotes the progression of HCC.

METTL5, one of the extensively studied methyltransferases of 18srRNA, increases protein translation in cancer, decreases cell apoptosis, and enhances cell cycle progression.^28^ In addition, the latest research shows that METTL5 is also associated with cellular metabolism. It can regulate glycolysis and cell proliferation in HCC by modulating c‐Myc.^29^ In our investigation, we employed immunohistochemical techniques to examine prognosis and expression level simpact of METTL5 in HCC tissues. The findings revealed that the expression level of METTL5 was substantially lower in HCC tissues compared to adjacent tissues and exhibited a positive correlation with pathological stages. Concerning the influence of METTL5 expression on the overall survival rate of HCC patients, analyses of Kaplan–Meier survival and multivariate Cox survival were conducted, which demonstrated that overexpression of METTL5 predicted an unfavorable prognosis. As a result, METTL5 may play a critical role in the progression of HCC. Furthermore, proliferation, migration, and invasion of liver cancer cells were suppressed when METTL5 was inhibited, increased cell apoptosis, and consequently hindered liver cancer progression. A hypothesis validated through in vitro experiments has been that METTL5 could have a negative impact on HCC cell proliferation. In animal studies, it was also observed that the growth rate of tumors decelerated and tissue necrosis intensified following downregulation of METTL5. These outcomes propose that METTL5 could potentially serve as a novel marker for evaluating the prognosis of patients with HCC. At the same time, we have discovered that METTL5 in HCC may be involved in the regulation of tumor immunity, which has not been reported before.

Immune cells are critical components of the tumor microenvironment (TME), with neutrophils being the most abundant and playing a key role in tumor initiation and progression.^30^, ^31^ Neutrophil extracellular traps (NETs) are web‐like structures from activated neutrophils releasing protein and DNA–histone complexes, capable of capturing bacteria, fungi, viruses, and other pathogens.^32^, ^33^, ^34^ With the in‐depth study of NETs, the levels of NETs in the plasma of patients with different types of tumors, such as bladder, lung, and pancreatic cancer, are higher than those of healthy controls.^35^ The presence of NETs has been found in a variety of metastatic tumor tissues, it shows NETs can trap cancer cells and serve as adhesive substrates, promoting cancer cell metastasis.^36^, ^37^ In HCC, both NETs synthesis and release increase.^38^

In our study, we utilized RNA transcriptome sequencing to analyze downstream pathways after METTL5 downregulation. First, GO functional enrichment analysis revealed METTL5 downregulation significantly correlated with neutrophil function. Subsequent KEGG pathway enrichment analysis showed it could significantly affect NET formation and release. From this, we conjectured METTL5, besides its own ability to promote cancer cell proliferation and invasion, also regulates tumor immunity to further accelerate HCC progression. To verify this, we performed immunofluorescence staining on xenograft tumor model tissue sections, using Cit H3 and MPO antibodies,^39^ common markers for detecting NET formation. Tissue sections from transplanted tumors with downregulated METTL5 showed reduced Cit H3 and MPO expression, indicating reduced NET formation and release with decreased METTL5 expression. In a coculture system of HCC cells with low METTL5 expression and HL‐60 cells, downregulation of METTL5 reduced HL‐60 cell differentiation into neutrophils and production/release of NETs. Our experiments suggest METTL5 has an important regulatory role in promoting neutrophil activation and NET formation/release in tumor immunity.

NETs have been recognized as promising cancer therapeutic targets in current studies, and inhibiting NET formation and activity in cancer may be a viable option considering their role in promoting cancer cell proliferation, invasion, and metastatic potential.^40^ However, current studies have not elucidated optimal intervention against NETs, and our study suggests METTL5 may be an important target for influencing NET release, providing new ideas for NET intervention. However, we also noted deficiencies in this study. Our statistical analysis of METTL5 and clinicopathological information based on HCC immunohistochemistry microarrays revealed no statistical difference in distant metastasis probability between patients in the METTL5 high‐expression group and low‐expression group, which may be related to the small number of patients with distant metastasis included in the immunohistochemistry microarrays. In addition, limited clinical specimens prevented further validation of NET expression and release in HCC tissues. Finally, METTL5 promoted neutrophil activation and NETs release, but the specific molecular mechanism remains to be further investigated. Overall, the present study demonstrated that abnormally high METTL5 expression in HCC tissues enhanced the malignant biological behavior of HCC cells and further accelerated HCC progression by promoting NET formation and release, an independent factor affecting HCC prognosis. Our findings suggest METTL5 may be a potential prognostic marker and target for HCC immunotherapy, providing new insights for future targeting of METTL5 and inhibition of NET formation and release to treat HCC.

## 4 CONCLUSIONS

Hepatocellular carcinoma overexpresses the N6‐methyltransferase METTL5 which correlates with malignant cell behavior and poor prognosis. Furthermore, METTL5 overexpression may induce NET release, influencing the tumor immune microenvironment and promoting distant cancer cell metastasis. Accordingly, we suggest that METTL5 as an immunotherapeutic and prognostic marker is promising for HCC.

## AUTHOR CONTRIBUTIONS

**Qi Wang:** Project administration (equal); writing – original draft (equal); writing – review and editing (equal). **Yuxi Huang:** Validation (equal); writing – original draft (equal). **Yu Zhu:** Methodology (equal). **Wenlong Zhang:** Formal analysis (equal); funding acquisition (equal). **Binfeng Wang:** Funding acquisition (equal); resources (equal). **Xuefeng Du:** Funding acquisition (equal); software (equal). **Qiqiang Dai:** Funding acquisition (equal); resources (equal). **Fabiao Zhang:** Funding acquisition (equal); methodology (equal). **Zheping Fang:** Funding acquisition (equal); methodology (equal); project administration (equal); supervision (equal); writing – review and editing (equal).

## FUNDING INFORMATION

The research was supported by a grant from the Chinese National Natural Science Foundation (Grant No. 81872237), the Science Technology Program of Zhejiang Province on the Scientific Research Project (Grant No. LGF19H160018), the project of Zhejiang Provincial Department of Health (Grant No. 2024KY1786/2021KY403/2024KY1787), and Taizhou Science and Technology planning Project (Grant No. 22ywa03/21ywa12).

## CONFLICT OF INTEREST STATEMENT

There are no conflicts of interest among the authors.

## ETHICS STATEMENT

Approval of the research protocol by an institutional reviewer board: N/A; Informed consent: N/A; Registry and the registration no. of the study/trial: N/A; Animal studies: All animal experiments were approved by Taizhou Hospital's Animal Experimentation Ethics Committee.

## Data Availability

This study used open source data from UC Santa Cruz's Xena (https://xenabrowser.net/datapages/) and the TIMER 2.0 database (http://timer.cistrome.org/).
